# Practitioner perspectives on best practice in non‐treatment factors that support the delivery of repetitive transcranial magnetic stimulation (rTMS) for depression

**DOI:** 10.1111/jpm.12815

**Published:** 2022-01-12

**Authors:** Sharon Mallon, Kate Walker, Zana Bayley, Chris Griffiths

**Affiliations:** ^1^ Open University Milton Keynes UK; ^2^ Northamptonshire Healthcare NHS Foundation Trust Northampton UK

## Abstract

**What is known about the subject?:**

- The practices of mental health nurses in the administration of repetitive transcranial magnetic stimulation (rTMS) treatments for depression in outpatient clinic are crucial for patient outcomes
- To date, most research has focused directly on procedural aspects of treatment delivery with limited focus on the delivery of holistic care and treatment.
- There is a lack of best practice guidance based on the experiences of those involved in clinical delivery to inform and improve rTMS practices

**What this paper adds to existing knowledge?:**

- This study provides unique insights into service and personalized non‐treatment factors associated with rTMS delivery that may reduce stress and improve the experiences of rTMS patients
- It reviews and updates understanding of the factors that contribute to the delivery of effective rTMS.

**What are the implications for practice?:**

- The need to apply findings for the development of best practice guidance
- Factors to improve practice include (a) rTMS machine demonstrations; (b) constructive, individualized, friendly, and therapeutic conversations; (c) a relaxing, comfortable, 'homely' physical environment; (d) long term supportive management; and (e) careful engagement of nursing and support staff.

## 1 INTRODUCTION

Major depressive disorder (MDD) is a large contributor to global burden of disease (WHO, 2017). Depression carries a large cost to society through assessment, monitoring, care and treatment as well as the loss of productivity and societal contribution of those affected (Greenberg et al., 2015). Relapse rates of depression remain high (Huynh & McIntyre, 2008). Treatment‐resistant depression (TRD) is considered to be no response to at least two consecutive courses of antidepressant medication (Berlim & Turecki, 2007). Between 12% and 20% of depressed patients have TRD (Nemeroff, 2007).

Repetitive transcranial magnetic stimulation (rTMS) is a form of neuromodulation: a non‐invasive and non‐convulsive technique where a purpose‑made electromagnetic coil is placed against the patient's scalp. These deliver short, powerful magnetic field pulses that painlessly induce electric currents in the cerebral cortex in the conscious subject (Hardy et al., 2016). rTMS is recommended to treat depression by United States' Food and Drug Administration (FDA) (Janicak & Dokucu, 2015) and UK's The National Institute for Health and Care Excellence (NICE, 2015). NICE (2015) declared it safe and effective in reducing depressive symptoms compared to sham rTMS (mimicking rTMS procedures, the auditory and/or somato‐sensory effects of active rTMS without actual stimulation of the brain). NICE noted that reports from patients were positive: with significant benefits to their quality of life, including some who felt able to stop oral antidepressant medications (NICE, 2015). In the UK, there is limited availability in the National Health Service (NHS) but it is widely available through private healthcare providers. It is expected that demand for rTMS will increase as knowledge of its effectiveness becomes more widespread and patient demand increases.

Generally, psychiatric nurses' involvement is the key in rTMS experience (Rosedale, 2009; Rosedale et al., 2009). Nurses seek to ameliorate treatment application side effects and monitor the patient for seizure activity (Belmaker et al., 2003; Van Trees et al., 2017). During treatment, they assess if pain experienced is within the expected boundaries and engaging the patient in conversation can reduce psychological impact of pain (Van Trees et al., 2017). Effective post‐treatment follow‐up procedures are also valuable in shaping patient's experience, if for example the patient reports worsening depression or mania they receive further evaluation by a psychiatrist (Van Trees et al., 2017).

To date, there has been a lack of research exploring “non‐treatment” factors in rTMS. Qualitative rTMS research has been lacking, a systemic review only found four articles (Health Quality Ontario, 2016). Only one of these, (Rosedale, 2009; Rosedale et al., 2009), used in‐depth interviews with patients, and these were US‐based participants drawn from a larger research study. Qualitative rTMS studies have focused on patient's experience of the treatment itself; rather than the engagement, information, support, nursing care or follow‐up package used in the delivery of rTMS. Currently, very little is known about individual care and support practices (Van Trees et al., 2017). Expanding this knowledge is likely to be beneficial to both rTMS patients and treatment providers (Rosedale, 2009; Rosedale et al., 2009). Patients are awake during the procedure and thus their experiences are important.

Limited procedures and guidance for rTMS service delivery, and specifically the role of a psychiatric nurse, were outlined as rTMS began to be used clinically (Bernard et al., 2009). However, there is a lack of best practice guidance based on experiences of those involved in clinical delivery to inform emerging rTMS practitioners and improve the practice of those already established (Bernard et al., 2009; Rosedale, 2009; Rosedale et al., 2009; Van Trees et al., 2017). In the absence of informed best practice guidance, it is possible that the treatment experience is less positive, patient's stress is higher and consequently treatment response and remission rates are lower. Delivery undertaken by private healthcare providers may adopt an approach minimizing costs and maximizing profits by limiting levels of patient–clinician interaction, opposing recommended practice (McClintock et al., 2017). This project sought to gain the views of staff delivering rTMS in the UK's NHS on aspects of care they think enhance or detract from rTMS experience. The aim was to understand and explore the non‐treatment factors associated with service delivery that staff report may reduce stress and improve patient experiences for those who receive rTMS on the NHS. The intention is that these can be developed as other rTMS services emerge.

## 2 METHODS

The study gathered data from seventeen members of staff working in NHS trust settings in England. All sites delivering rTMS in the NHS (*n *= 4) were contacted. All staff involved in the procedure at those sites who agreed to take part (*n *= 3) were given a letter by their line manager asking them to contact the researchers if they were interested in participating in an interview about rTMS delivery. Their professions have been presented in Table 1. Most participants were female (*n *= 15). The interviewing team comprised two experienced qualitative researchers (Authors 2 and 3) one of whom works in the HE sector, and one of whom is a contract researcher within a NHS setting not connected to participants. Both authors have PhDs and prior research in healthcare settings. None of the authors have any personal experience of, or investment in, the delivery of rTMS beyond the aims of this study.

**TABLE 1:** Professional title of participants

Professions	Professional Context
Consultant	Consultant in Adult Psychiatry (hospital based) (*n* = 2)
Nursing staff	Associate Director, Inpatient Emergency Care & rTMS Manager of Services (*n* =1) Student nurse (*n* = 1) Matron (*n* = 1) Registered Mental Nurse (*n* = 4) Healthcare Assistant (*n* = 3)
Researchers	Research Assistant rTMS study (neuropsychology/psychology) (*n* = 4)
Non‐clinical	Administrator/receptionist rTMS clinic (*n* = 1)

### 2.1 Data collection

Information was gathered using semi‐structured interview guides, consisting of open questions formulated around key topics and prompts drawn from existing rTMS literature. These included guidance and information provided to patients, interactions during and post delivery, physical environment and actions to reduce patient stress. The interview guides sought to obtain views from staff in relation to the practical aspects of rTMS delivery but allowed staff to determine the overall direction of the interview. At the request of the participants, most of the interviews were undertaken by phone (*n* = 15), with two taking place individually within private rooms in the hospital setting. They lasted between 20 and 45 min and were digitally recorded and transcribed by the interviewers and checked by Author 1. Interviews were concluded when Authors 1, 2 and 3 agreed that data saturation had been achieved.

### 2.2 Analysis

Interview data were analysed by Authors 1 and 3 using thematic analysis (Braun & Clarke, 2006). The analysis focused on the development of a coding framework initially driven by the original research objectives and by emergent themes from the literature. However, these themes were then shaped by team discussion of the pertinent issues that developed during the interviews and by repeated readings of the transcripts to identify those issues emerging from participants. Initially, researchers involved in data collection read through transcripts focussing on key topics, from this preliminary work a coding framework was developed. Following this segmentation of the data, a process of interpretative analysis was undertaken with researchers discussing themes (Miles & Huberman, 1994). Following this, themes were reviewed to ensure validity both against the literature and within the data. Nvivo was used to store transcripts and to capture thematic analysis.

### 2.3 Research findings

rTMS treatment has a procedural flow that is largely determined by operating procedures. Thus, thematic findings are presented in chronological order, from the early engagement with patients to post‐treatment. The findings are reported under five headings: “pre‐treatment visit”; “communication and rapport”; “environment”; “aftercare”; “staff morale.” It is worth noting, that while our questions anticipated procedural elements would dominate, the responses, as illustrated below, demonstrated that staff were focused on the experiential interpretation of these processes from the perspective of the patient.

### 2.4 Pre‐treatment Visit

This section explores pre‐treatment procedures. The centrality of these processes was not something we had anticipated in our interview schedule. This theme emerged in response to the issues highlighted by staff. In all cases, the rTMS procedures included an introductory patient visit. Everyone interviewed mentioned printed support material provided to patients prior to treatment. This included specific information relating to treatment, travel, parking and access. The core part of the pre‐treatment visit was rTMS machine demonstration.

#### 2.4.1 Machine demonstration

The visit involved showing patients the clinical setting, including the equipment. This “walk around” had a dual purpose: make the patient feel welcome and alleviate anxieties prior to treatment. Staff explained the printed information and various aspects of the procedure:

…, if you sit in a room with them with the machine, I can explain all this [the treatment] to them again2026 what you don't realise is that somebody's very, very nervous (9BW).

It allayed concerns about treatment delivery; challenging any presumptions that patients may have

Taking someone into a room, showing them the equipment, showing them how it works, … hopefully alleviating some anxieties really, because I think people that have got thinking they're having a magnet on their head have got all weird and wonderful misconceptions about how that might look and feel…(3BW).

Most cases included a treatment trial:

“They can have a look around if they want, they can see the machine in action, sometimes people get worried by how noisy it is, or how painful it is, so sometimes they have a go on their arm so they can feel the sensation to work out if they can tolerate it or not” (1BW).

Where the patient was initially reluctant to do this, staff were willing to demonstrate the machine on themselves:

I let them feel the coil before they actually put it on their head, and if they feel anxious and worrying and thinking it's going to be really horrible, I always put my hand under so it gets me, so they can see I’m not frightened of it, and then they can say ‘oh yeah you're doing it’ so it's just to show them that there is nothing to fear, so try and take the anxiety away (7BW).

Staff described this step as being crucial in seeking informed consent:

Obviously in order to get the written consent they have got to understand what the treatment is about so we have to do all that kind of sense checking and making sure they have understood the information that has been given to them (KW3).

Staff reported that it was important to individualize their responses:

Not kind of having stock answers or just like giving them a quick explanation, but actually taking the time before we started treatment to just like sit down, … properly talk through any anxieties they have about it. (KW7).

#### 2.4.2 Initial reassurance

The purpose of the pre‐treatment meeting was described as offering encouragement and removing any anxieties. Consent was a significant decision for patients, and they were often unsure. Staff felt it was important that reassurance commenced prior to service visit:

We'll get patients who, if we are booking appointments for them and they are quite anxious about getting their treatment started, things like that, so a lot of reassurance there, that they're doing the right thing, sometimes they are apprehensive about starting and what it involves (5BW).

Early encounters were used to gain information that helped the team to personalize the approach taken; for example, by tailoring the timing of appointments:

We get patients that struggle to get out of bed in the morning because they are so depressed, for example, and so morning appointments aren't going to be suitable for them, so it's little things like that, that show we care about them… (5BW).

The appointment schedule provided at the end of the visit was seen as a crucial part of reassurance:

It's typing up their appointment lists as well because as you can imagine you are booking them in every day, so if you typing up their appointment lists, they've got it there and they know where they are at, and it's an extra kind of comfort for them… (5BW).

### 2.5 Communication and rapport

The pre‐treatment visit presented an opportunity to reassure the patient prior to treatment. This communication represented the first step in ongoing reassurances throughout their visits, from welcoming and personalized greetings by reception staff, to conversations about the procedure.

#### 2.5.1 Ongoing reassurance at treatment outset

Managing the anxieties of patients was at the core of communication during the first treatment delivery session. This potentially included delaying or stopping treatment:

We wouldn't start treatment until they feel comfortable, if there are any anxieties, and sort of tearfulness then we wouldn't start or if that had started, if we'd commenced the treatment, we'd stop the treatment (3BW).

This was monitored on a daily basis, as patients could be in an emotional state and treatment could not be started:

A lady was too distressed to receive her treatment, so … you know that is priority, we want to administer that treatment today but we can't if she is feeling that upset (3BW).

Some patients became upset during the treatment delivery; basic acts of comfort such as holding hands or chatting were sometimes sufficient to ensure patients were able to continue:

We go and sit with them, sometimes we'll hold their hand and you know just comfort them basically, and just chat to them, whether that be if we've stopped the treatment or throughout the treatment if they don't want to stop the treatment… (8BW).

Although staff were mindful of the technological aspects of successful treatment delivery, they sometimes stopped the delivery of treatment. Their approach reflected that, although rTMS is a mechanistic procedure, it was important to ensure that the patient was at the centre of decisions made about the treatment. The responsiveness of staff was key to ensure that patients’ needs were met:

Some of them want to close their eyes and just relax through it and others want to talk, so it's, we make it about them. So everything that they want then I try to meet that need2026 (KW5)

#### 2.5.2 Purposeful daily chats

All staff reported that a great deal was gained from patient communications and building rapport:

I think it's how you present yourself, whether they can feel that they can open up to you, some people are really, really quiet, you get no eye contact, they don't want to talk to you, they don't want to look at you, they are holding in so you gently enable them to come out in their own time (7BW).

For some staff, patient therapeutic benefit came from being listened to:

We have had patients come in and say 'You do a great job, just talking to somebody that listens, that really does help' (6BW).

Some reported that benefit was gained by opening up during rTMS treatment, providing an alternative avenue for expressing feelings:

They talk about things that they can't talk about with their families because the families are fed up with hearing it, it's like a broken record, sometimes they feel like they are letting everybody down… (7BW).

Other staff considered interactions to be guided by specialist knowledge of counselling or CBT techniques:

In the course of talking to them we are counselling as well, and doing… cognitive behaviour therapy in a moderate form (2BW).

Regardless of specific techniques, most felt that, over the treatment course, patients were able to open up, enabling greater insights into their difficulties.

Documenting interactions that took place during treatment was an important part of the delivery of rTMS, as this information was used as part of patients' progress reviews:

… we ask them how they are, how they've been feeling, what their days consist of, have they done anything different to what they usually do, you know, has their thinking changed (8BW).

This was felt to bring additional qualitative insight into how, if at all, the treatment was helping alleviate symptoms. It provided a useful adjunct to the quantitative data gathered using psychometric ratings scales. Staff reported that, as the weeks went by, a sense of rapport grew between themselves and patients, improving progress assessments:

I find that the rapport we have is very good, so if I saw them yesterday and they tell me they are doing something the next day, I ask them 'how did it go?' sort of kick starts the conversation (4BW).

Rapport enabled patients to discuss aspects of treatment that they had previously not understood, adding to their feeling of safety:

And then they start to open up and then once they feel comfortable, they say 'Can you just tell me what this is, and what this is because I hear people talking about it and I haven't got a clue what it is'. So I think it's that confidence thing in the person and the staff here, and that feeling of safety (3BW).

#### 2.5.3 Reassurances about progress

Affirming that the patient was progressing was a feature of daily communications:

When someone is doubting themselves, they'll give a different perspective on it and saying ‘you might not think it but you are doing really, really well,’, you know celebrate the little successes (3BW).

Sometimes commenting on the achievement of making it to the treatment:

You can see the difference, yeah, people that don't want to get out of bed, but it's an achievement to come here… (8BW).

Mention was made of the need to support patients in relation to the impact of the treatment on their emotional state and the management of emotions:

So they've been quite numb, and these emotions suddenly surge and it can take a dip in mood, we reassure people with this, that this is actually the treatment working (9BW).

If the treatment was working well, this could be accompanied by a surge of positive emotions that the patient may not have experienced for some time. In these cases, patients needed to be encouraged to find ways of managing and employing these emotions:

… talking through with them, explaining why this has happened, and that this is a good thing, explaining what would happen next, coping strategies during this period (9BW).

### 2.6 Environment

This theme focused on the environment where the treatment takes place; including physical aspects and how the environment was emotionally perceived by patients.

#### 2.6.1 Physical environment

The physical environment in the clinic was adapted to enhance their treatment experience. Modifications included consideration of lighting, seating, temperature and room aesthetics. It included practical needs such as refreshments and toilet facilities, including those for families and carers:

it's very comfortable, it's warm, there's a little toilet pod for them, there's a little light, we turn lights down, there's a radio, there's.. stress balls! There's blankets, there's music… (9BW).

Aesthetically, staff made the space less like a hospital environment, making the room “bright and calm” adding additions such as wallpaper, pictures and ornamentation:

We got pictures on the wall to try and make it less like a hospital and more like somewhere pleasant, we even buy silly things like wiggle pots that just, they're solar powered and it's like a bumblebee that wiggles and takes their mind off it, something else to see, something different (7BW).

Some units had a television in the room with subtitles to allow the patient to watch despite the rTMS machine noise. The temperature of the room was carefully monitored, with an air‐conditioner if necessary.

A great deal of effort was made by staff to ensure the treatment chair was comfortable. This included repeated checks throughout the session, as some patients found sitting without moving caused discomfort. Patients were encouraged to bring in their own blanket or other items such as pillows or cushions that may provide them with comfort during the procedure. This was about letting the patient be in charge of their own environment, to create a feeling of safety: *the interaction really needs to match what the person actually wants*. (KW5).

#### 2.6.2 Emotional environment

Staff felt creating the right emotional atmosphere within the clinic was necessary. This was undertaken by creating an environment where patients felt listened to, safe and a sense of trust.

…feeling of safety, comfort, being able to talk and listened to, being heard, having a cup of tea, a carer being welcomed… (3BW).

A key aspect of creating the right emotional environment for effective treatment was related to staff continuity. Daily chats helped staff to build up a clear picture of the treatment response. Staff thought patients valued having consistency throughout treatment:

If they know a familiar face its putting them at ease, yeah, feel safe and comfortable because they will say that, that they feel safe with a particular person… (8BW).

### 2.7 Aftercare

Typically, aftercare involved phone calls after seven days. However, while there was a degree of standardization discussed by staff across the interviews, there were varying practices in relation to longer term aftercare. Staff expressed that some patients found ending their treatments difficult because they had built up a rapport with the team and felt part of a treatment “community.” In response to this, one clinic co‐ordinated a face‐to‐face patient support group:

Some patients will feel a lot better and will just go and get back to their normal routine, other patients are tearful and upset to be leaving and they will usually be pointed in the direction of the support group because they find it so pleasant coming here (5BW).

This group provided an opportunity for patients to talk about their experiences, meet others and maintain a connection with the clinic:

We have a monthly programme where certain patients come once a month, so they don't feel as though they are being chucked to the elements (7BW).

Clinics offered an informal and periodic review. In terms of continuing patient connection, most patients were advised they could contact the clinic at any time. All clinics contacted patients shortly after the end of treatment to check on progress, providing staff with the opportunity to assess if the patient was becoming depressed and might require “top up” treatments. This offer was considered important to provide reassurance:

There is the possibility of top ups and sometimes that alone is just enough to give that person reassurance that they are not walking out this building and they are on their own. (3BW).

### 2.8 Staff morale

Staffing of the clinic, experience and training of the team and team moral were not items on our original interview schedule but emerged from our analysis. Staff were positive about their working environment and outcomes of the treatment:

You do see the difference in them quite a lot, particularly from where they started and when they finish and it kinds of makes them feel better about what you are doing, and I think that kind of gives you enthusiasm to keep that level of service up for them (5BW).

This motivation was linked to rTMS service, which was contrasted with other nursing roles they had been involved in:

You see on the wards you get these people back all the time, you're not doing anything for them. Here, if 40% are skipping and laughing out the door, how good is that? (9BW).

There was a strong emphasis placed on the knowledge, experience and training of the staff. Notably, specific training or guidance for the rTMS clinic was not considered to be the most important. Most staff recalled training and skills received as a mental health nurse as being the skills that were more useful:

Most of us seem to be transferring our health experience as a nurse and a technician, some of them are 12, 20 years as a HCA and they've been talking to people and counselling them and all that, so it's like transferring our experience to use as well and its working (2BW).

There was a strong sense of the value in staff supporting each other; for example, by checking on each other during the treatments. The support they felt that they received from management contributed to feeling like a valued team member:

I think one of the things with managers from our team…and they actually treat you as an individual person… here you've got a voice, you've got an opinion and you're allowed to air it (7BW).

Our leaders are innovative and take notice of whatever staff want you to know to improve the service, and so staff are happy and ready to go that extra mile (2BW).

## 3 DISCUSSION

The aim of this study was to understand and explore non‐treatment factors associated with service delivery that reduce stress and improve outcomes for rTMS patients. As outlined below, aspects of these findings align with previous US research on patients' perspective, but our results add important context and findings from within the UK setting and update our understanding of delivery over 10 years after many of the original studies into this area were conducted.

This study found that staff intensively engaged with patients across the whole treatment journey. This included thorough information sent, and direct conversation prior to, during and after treatment. Allowing patients to learn about the rTMS machine through demonstrations was considered essential to the patient experience. It is consistently reported, here and elsewhere, that staff relationship and communication influenced treatment experience, completion and outcomes (Rosedale, 2009; Rosedale et al., 2009). Our data demonstrated that patients continue to value the therapeutic relationship with rTMS service staff and that this relationship was strengthened when time was allotted for conversations related to rTMS (Rosedale, 2009; Rosedale et al., 2009). Constructive, individualized, friendly and therapeutic conversations were key to effective treatment delivery, and staff continuity was considered important. Our research also shows the value of setting out clearly the appointment schedule at the outset of treatment. These findings add to the research evidence and are valuable in mental health nursing practice by highlighting how a holistic approach to the delivery of a highly technical procedure has been considered to be beneficial to patient experiences, from the perspective of staff who have developed these innovative services.

It has been noted that best practice protocols should be informed by patients’ experience to optimize treatment tolerability (Rosedale, 2009; Rosedale et al., 2009), as relayed by patients and staff working with them. Nurse‐managed services provide clinical efficiency, safety and patient satisfaction for a wide variety of diagnoses (Coddington & Sands, 2008; Krothe & Clendon, 2006). Psychiatric nurses have a unique skill set that makes them essential in the delivery of safe, effective and comfortable rTMS treatment (Van Trees et al., 2017). Through administering care and treatment encompassing the nursing metaparadigm and assessing outcomes, nurses continuously improve the quality of rTMS service delivery (Bernard et al., 2009).

Communication and an effective rapport allowed collation of extensive supportive information about a patient's progress, as well as personalized support throughout and following treatment. Additionally, our study also showed that nurse's engagement brought additional qualitative insight into how treatment was impacting on patient symptoms. In the absence of effective best practice guidance that details some of these qualitative elements of care, it is possible that the treatment experience could be less positive, patient's stress is higher and consequently treatment response and remission rates are lower. There remains a lack of practitioner informed best practice guidance for rTMS, the findings of this present study, in combination with existing evidence can begin to inform their development should services expand or seek to develop new sites.

Crucially, our experiences of collecting data for this study showed that while it was common for staff to reflect upon how complex and specialized their involvement was, when interviewing them it was also apparent how they took many informal procedures for granted. In some cases, only seeing their significance when asked to reflect upon them in a research interview. One aspect of care highlighted repeatedly by staff in our study was the importance of providing a relaxing, comfortable and ‘homely’ physical environment. The centrality of these factors in contributing to the patients’ positive experience had not been anticipated, has been poorly reported elsewhere and highlights the value of the holistic approach taken by mental health nurses. Additionally, this study adds to our understanding of the implications of good practice on staff morale and how this can contribute to strong engagement and motivation to deliver high‐quality care.

The findings here link to, and provide further insight into, the best practice application of phases in the nurse–patient relationship identified by Bernard et al. (2009): initial contact, evaluation and orientation, administering, treatment, providing case management and milieu management. This study found that developing a positive emotional environment which facilitated time for being listened to and a sense of trust is vital; this links to increasing the tolerability of treatment and improved outcomes (Bernard et al., 2009). A humanistic approach to the care of rTMS patients is valuable (Rosedale, 2009; Rosedale et al., 2009) and ongoing communication is key to providing effective care (Bernard et al., 2009). As rTMS demand grows and services upscale, it is important that these qualitative aspects of care are documented and continue to be implemented as their centrality to outcomes has been continued to be acknowledged and reinforced by this study.

### 3.1 Implications for mental health nursing practice

Incorporating this study's findings, best practice guidance for the implementation of non‐treatment related rTMS delivery should be developed to ensure that all aspects of the rTMS procedure are carried out in ways that maximize patient experience. This study reinforces that crucial aspects of care include physical setting, strong communication prior to and throughout the delivery of treatment. In addition, those expanding or setting up new rTMS treatment services should ensure staff are consulted and given appropriate responsibility and support, as high staff morale improves staff engagement with patient experience and may enhance positive outcomes.

### 3.2 Study limitations

Study limitations include the relatively small sample size, but this is considered an appropriate number for qualitative in‐depth interviews (Guest et al., 2006). There is also a potential lack of generalizability associated with the qualitative methodology as those who agreed to participate were self‐selected and there may be some bias in terms of those agreeing to participate having a more positive view. The direct voice of the patient is absent from this current research, although it is represented indirectly in some of the information given by staff interviewed. The research was conducted in the National Health Service (NHS) in the UK, but it is to be noted that the UK is a multicultural society. The study did not include private providers of rTMS.

## 4 CONCLUSIONS

Maximizing the positive experience and response rate, and minimizing stress and remission following rTMS is a goal of clinical services. The findings and recommendations of this study have the potential to enhance the value of treatment to patients and contribute to treatment effectiveness. The influence and impact of non‐treatment contributory factors, which surround the treatment and how these combine with the actual treatment, needs to be better evaluated if the best possible outcome for patients is to be achieved. It is not fully understood the degree to which non‐treatment factors reduce stress and if these may contribute to improved response rate and remission, research could be undertaken to test this. Future research comparing NHS and private rTMS service delivery to identify difference and similarities and reasons for these could be justified. Given the importance of these procedures, it is also vital that future research places service user voices at their core. This should be through both consultation during the research design process and if appropriate qualitative interviews to collect the experiences of mental health service users who have undergone this procedure.

## 5 RELEVANCE STATEMENT

This provides unique insights into the personalized service delivery of rTMS treatment in the UK context. It details factors contributing to existing research on the delivery of rTMS. These factors include (a) rTMS machine demonstrations; (b) constructive, individualized, friendly and therapeutic conversations; (c) a relaxing, comfortable, “'homely' physical environment; (d) long‐term supportive management; and (e) careful engagement of nursing and support staff. These update and add to previous research related to practice guidance (Bernard et al., 2009; Rosedale, 2009; Rosedale et al., 2009; Van Trees et al., 2017).”

## CONFLICT OF INTEREST

All authors declare that they have no conflicts of interest.

## AUTHOR CONTRIBUTION

All authors contributed to the design of the study and interpretation of results. All co‐authors contributed to critically revising the manuscript, read and approved the final manuscript and agree to be accountable for all aspects of the work.

## ETHICAL STATEMENT

The study was approved by UK's Health Research Authority (HRA) Research Ethics Committee (REC) and The University (Name removed for review) ethics committee.

## Data Availability

The data that support the findings of this study are available on request from the corresponding author. The data are not publicly available due to privacy or ethical restrictions.
